# Interaction Among Microbiota, Chemical Composition, and Quality Variations in *Coffea arabica* Across Altitudinal Gradients During Wet Processing

**DOI:** 10.1002/fsn3.72145

**Published:** 2026-07-30

**Authors:** Xiaojing Shen, Yamei Wu, Qi Wang, Wenhua Chen, Bintao Peng, Surui Lu, Ying Yang, Kunyi Liu, Jilai Zhang

**Affiliations:** ^1^ College of Science & College of Resources and Environment & College of Architecture and Engineering Yunnan Agricultural University Kunming Yunnan China; ^2^ School of Wuliangye Technology and Food Engineering Yibin Vocational and Technical College Yibin Sichuan China; ^3^ School of Resources and Environment Baoshan University Baoshan Yunnan China

**Keywords:** chemical compounds, *Coffea arabica*, coffee flavor, microbial diversity, planting altitude

## Abstract

Planting altitude is a critical environmental factor shaping the flavor quality of *Coffea arabica* . However, how altitude influences flavor through microbial dynamics and chemical transformations during wet processing remains poorly understood. This study systematically investigates microbial diversity during wet processing and the corresponding changes in non‐volatile and volatile compounds in coffee beans from four elevations: 1000 m (A1), 1200 m (A2), 1400 m (A3), and 1600 m (A4). The results showed that fermentation significantly altered the dominant bacterial genera from *Sphingomonas* and *Pleomorphomonas* (before fermentation) to *Weissella* and *Lactobacillus* (after fermentation), while *Cladosporium* and *Fusarium* remained the dominant fungal genera. Notably, non‐volatile compounds in de‐pulping coffee beans and volatile compounds of roasted coffee beans showed significant differences. Among them, 57 (1000 m), 218 (1200 m), 133 (1400 m), and 173 (1600 m) differentially changed non‐volatile compounds (DCn‐VCs) belonging to lipids and lipid‐like molecules, organic acids and derivatives, organoheterocyclic compounds, organic oxygen, phenylpropanoids and polyketides, benzenoids, and other classes were identified. The cupping score of roasted coffee beans increased with increasing planting altitude, and A4 exhibited a long‐lasting aftertaste, mellow and full body with high sweetness, nutty, and flowery with 42 (A4 vs. A1), 27 (A4 vs. A2), and 26 (A4 vs. A3) volatile compounds showing significant variation across altitudes. Therefore, planting altitude significantly influences coffee flavor by reshaping microbial diversity and chemical composition during wet processing. High‐altitude cultivation promotes desirable sensory characteristics, highlighting its potential for producing specialty‐grade coffee.

## Graphical abstract

Fermentation significantly altered the dominant bacterial genera from *Sphingomonas* and *Pleomorphomonas* (before fermentation) to *Weissella* and *Lactobacillus* (after fermentation), while *Cladosporium* and *Fusarium* remained the dominant fungal genera. Notably, non‐volatile compounds in de‐pulping coffee beans and volatile compounds of roasted coffee beans showed significant differences. Among them, 57 (1000 m), 218 (1200 m), 133 (1400 m), and 173 (1600 m) differentially changed non‐volatile compounds (DCn‐VCs) belonging to lipids and lipid‐like molecules, organic acids and derivatives, organoheterocyclic compounds, organic oxygen, phenylpropanoids and polyketides, benzenoids, and other classes were identified. And the cupping score of roasted coffee beans increased with increasing planting altitude, and samples of 1600 m exhibited a long‐lasting aftertaste, mellow and full body with high sweetness, nutty, and flowery.

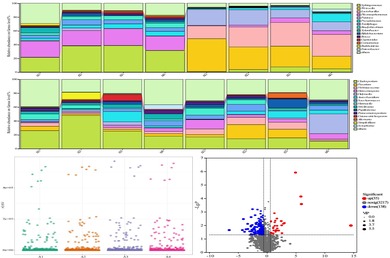

## 1 Introduction

Coffee is originally from the tropical forests of Ethiopia. There are 131 species in the genus *Coffea*, which is widely distributed and adapted to tropical and subtropical regions (Lyrio et al. 2025; Cassamo et al. 2023). Among beverages, coffee enjoys one of the highest consumption rates across the globe, prized for its distinctive aroma and flavor, and represents a major agricultural commodity with significant implications for international trade, tropical agriculture, and the livelihoods of people worldwide (Wang, Liu, et al. 2025; Wang, Quan, et al. 2025; Cassamo et al. 2023). The statistics data showed coffee provided an economic income to more than 25 million families from more than 80 planted countries (Wang, Liu, et al. 2025; Wang, Quan, et al. 2025; Cassamo et al. 2023). In recent years, the global coffee sector has witnessed substantial expansion, with the ICO Composite Indicator Price (I‐CIP) averaging 334.41 US cents per pound in May 2025—an increase of 60.5% compared to May 2024 (https://ico.org/).

Besides its economic significance, coffee also offers numerous health benefits due to its multiple biological activities attributed to its antioxidant and anti‐inflammatory compounds, including caffeine, chlorogenic acids, and diterpenes (Kunutsor et al. 2025). Moderate intake of coffee has been linked to enhanced longevity through reduced mortality related to cardiovascular, cerebrovascular, cancer, and respiratory diseases, while also alleviating major contributors to age‐related functional decline (Lopes and Cunha 2024). It has also been associated with reduced risks of skin, liver, prostate, and endometrial cancers by mitigating oxidative stress, inhibiting cancer cell proliferation, inducing apoptosis, and modulating hormone levels (Kunutsor et al. 2025). Moreover, coffee intake exerts beneficial effects on both oral and gut microbiota, as well as gastrointestinal motility (Saygili et al. 2024). Beyond the well‐documented contributions of caffeine, chlorogenic acids, and diterpenes to human health, coffee is also a significant dietary source of essential and nonessential mineral elements; for instance, a comprehensive analysis of both Arabica and Robusta varieties determined the concentration of 15 major and trace elements, including K, Mg, Ca, P, S, Na, Fe, Mn, Cu, Zn, and Cr. While the contribution of a single serving to the recommended daily intake of essential elements may be low, this contribution is significant when considering that coffee is a beverage consumed daily by billions of people worldwide (Senila et al. 2025).

*C. arabica* and *C. canephora* represent the two primary coffee cultivars, accounting for 99% of the green coffee bean production in the world (Lyrio et al. 2025). According to the United States Department of Agriculture (USDA), the 60‐kg bag yields have reached 76,380 and 99,855 for Robusta and Arabica coffee, respectively, with a projected increase of 7.1 million bags for the 2024/25 season. Native to Ethiopia and Yemen, *C. arabica* thrives in humid, warm environments between 500 and 2400 m above sea level, with optimal growth typically occurring at 1000 and 2000 m (Diaz‐Gómez et al. 2025). Now, Arabica coffee is mainly produced in Brazil, Colombia, Ethiopia, and Honduras, accounting for approximately 70% of total coffee production worldwide, with a smooth, rich flavor profile, delicate flavor, and aromatic qualities (Hong et al. 2024). Its successful cultivation requires precise agroecological conditions, notably elevations between 600 and 2000 m above sea level. Cooler climates, adequate rainfall, and well‐drained soil create optimal growing environments, while natural shade from canopy trees and a biodiverse ecosystem further enhance plant vitality and flavor complexity (Abdelwahab et al. 2024).

Ranked just below water in terms of global consumption and trailing only crude oil in trade volume, coffee holds substantial commercial value, largely driven by its complex and desirable flavor profile (Hu et al. 2020). In response to growing consumer interest in specialty coffee, Arabica coffee has gained widespread popularity (Abdelwahab et al. 2024). However, its quality is significantly shaped by ecological variables including altitude, temperature, humidity, and soil composition (Diaz‐Gómez et al. 2025). The planting altitude is essential for flavor development, influencing the formation and accumulation of coffee flavor precursor substances (such as lipids, sucrose, proteins, chlorogenic acid, caffeine, and trigonelline) that undergo differential accumulation in green coffee beans. For example, the levels of alkaloids and chlorogenic acids declined at higher altitudes, while the content of fatty acids increased. These coffee flavor precursor compounds not only can influence coffee sensory but also can form flavor compounds to produce aroma‐active compounds during roasting (Lee et al. 2015). At the same time, the coffee cultivation altitude substantially influenced several volatile substances, increased the caramel and sweet sugary flavors, and decreased the roasted and nutty aromas (Hu et al. 2024). Moreover, altitude affects the biochemical attributes of ecosystems, such as acidity and antioxidant capacity, further influencing quality and yield (Martinez et al. 2022; Martins et al. 2020; Haro et al. 2025).

Globally, coffee is cultivated across equatorial regions with frost‐free climates, adequate rainfall, and well‐drained soils (Han et al. 2025). In China, coffee cultivation dates back to 1884, with large‐scale planting beginning in the early 20th century in Binchuan County, Yunnan Province (Han et al. 2025). Today, Yunnan is China's premier coffee‐growing region, contributing over 98% of the national output and cultivation area. Major production zones include Puer, Baoshan, Dehong, Lincang, and Nujiang municipalities (Hu et al. 2025; Zhai et al. 2024). In 2024, Yunnan produced 1,496,000 tons of coffee across 1.267 million mu of land (Chen et al. 2022). Due to its advantageous geographic location and favorable climatic conditions, Yunnan Arabica coffee is internationally recognized for its unique flavor and high quality, characterized by a sweet aroma and aromatic acidity (Ma et al. 2022).

With increasing global demand, China's coffee industry has witnessed substantial growth in production and trade (Zhai et al. 2024). Against this backdrop, the present study investigates how planting altitude influences the flavor quality of wet‐processed Arabica coffee beans. The focus is on characterizing changes in non‐volatile and volatile compounds to support the development of the coffee industry as one of the important agricultural economies.

## 2 Materials and Methods

### 2.1 Materials

Fully ripe red coffee cherries ( *C. arabica* ) with uniform size, color without visible disease and mold at elevations of 1000, 1200, 1400, and 1600 m above sea level were hand‐picked on March 2025 in Baoshan City, located in the southwestern Chinese province of Yunnan. Three samples collected at each altitude, which were marked A1, A2, A3, and A4, respectively. Once harvested, coffee berries stored in aseptic bags were transported to a laboratory (Kunming, Yunnan province) within 8 h for wet processing to obtain green coffee beans.

HPLC‐grade methanol (CAS 67‐56‐1), acetonitrile (CAS 75‐05‐8) were purchased from Thermo Fisher Scientific (Pittsburgh, USA), formic acid (CAS 64‐18‐6) from Shanghai Anpel Experimental Technology Co. Ltd. (Shanghai, China), and 2‐propanol (CAS 67‐63‐0) from Merck Inc., respectively. Water (7732‐18‐5, LC–MS grade) was purchased from Thermo Fisher Scientific (Pittsburgh, USA). E.Z.N.A. soil DNA Kit (Omega Bio‐tek, Norcross, GA, USA). A Waters ACQUITY UPLC HSS T3 column (100 mm × 2.1 mm, 1.8 μm, Milford, USA) using an UPLC‐MS/MS system (UHPLC‐Triple TOF 6600, AB SCIEX, USA) was used to the analysis of non‐volatile compounds in various coffee bean samples. While, the mixture of C_8_–C_20_ n‐alkanes standard mix solution (40 mg/L each, in hexane) were purchased from Sigma‐Aldrich (Shanghai, China), a headspace solid‐phase microextraction coupled with gas chromatography–mass spectrometry (HS‐SPME‐GC–MS) (8890‐7000D) system, equipped with a DB‐5MS capillary column (30 m × 0.25 mm × 0.25 μm), was purchased from Agilent Technologies (Santa Clara, CA, USA) for the analysis of volatile compounds in various roasted coffee bean samples.

### 2.2 Sample Preparation

The first step was the operation of sorting and floating to remove the overripe, dried, and underdeveloped coffee cherries. Then, the coffee skin and pulp surrounding the coffee seeds were removed by hand‐picking and de‐pulping. Subsequently, natural fermentation under a natural environment was operated in an open environment under room temperature ranging from 10°C to 22°C and humidity from 41% to 43%, in which 600 g of de‐pulping coffee beans were mixed with 900 mL H_2_O (the pH was 6.8, the content of dissolved oxygen was 0.17 mg/L). During the wet processing, coffee samples at two fermentation time points at 0 h (planting altitude samples marked A11, A21, A31, and A41) and after 48 h (planting altitude samples marked A12, A22, A32, and A42) were collected and stored at −20°C until needed for Illumina‐based amplicon sequencing and UPLC‐MS/MS analysis. Following fermentation, the coffee beans underwent washing and sun drying until moisture levels reached 12% to acquire green coffee beans, which were subsequently roasted to a medium‐roasted degree using an IKWA Pro V3 coffee bean roaster (IKAWA Ltd., London, UK) under 10–15 min roasting time, 220°C roasting temperature to obtain brownness roasted coffee beans. Finally, the roasted coffee beans were then evaluated using HS‐SPME‐GC–MS profiling.

### 2.3 Microbial Diversity Assessment

Majorbio Bio‐Pharm Technology Co. Ltd., located in Shanghai (China), performed Illumina high‐throughput sequencing to carry out Illumina‐based amplicon sequencing of the coffee fermentation samples. DNA belonging to microbial genomes was isolated using the FastPure Stool DNA Isolation Kit (Magnetic Bead) provided by MJYH, located in Shanghai (China). Bacterial assessment involved employing the 1193R (5′‐ACGTCATCCCCACCTTCC‐3′) reverse primer and the 799F (5′‐AACMGGATTAGATACCCKG‐3′) forward primer for 16S rRNA gene amplification to obtain the sequences. Fungal ITS regions were amplified using the ITS3F (5′‐GCATCGATGAAGAACGCAGC‐3′) and ITS4R (5′‐TCCTCCGCTTATTGATATGC‐3′) primer pair. The PCR protocol began with a preliminary strand separation (95°C, 30 min), followed by 27 cycles of DNA strand separation (95°C, 30 s each), hybridization (55°C, 30 s), and a further 45 s at 72°C. Further strand synthesis was performed at 72°C for 10 min, concluding with a hold at 4°C. The second round of amplification was conducted over 13 cycles. Equimolar purified amplicon concentrations were sequenced using a paired‐end method on the Illumina Nextseq2000 sequencing system (Illumina, San Diego, USA). The resulting raw sequencing data were entered into the NCBI Sequence Read Archive (SRA) database (Accession Numbers: PRJNA1251849 and 1251855). Operational Taxonomic Units (OTUs) were clustered at a 97% sequence similarity threshold, which typically corresponds to the taxonomic level of species, serving as the minimum classification level for subsequent analyses. Alpha diversity indices, including Chao, ACE, Simpson and Shannon, were calculated to assess species richness and diversity within samples.

### 2.4 Non‐Volatile Compounds Analysis

UPLC‐MS/MS was served to examine non‐volatile compounds (n‐VCs) (Shen et al. 2025a, 2025b). Each analysis began with 150 mg of ground coffee mixed with 1.20 mL of an 80% methanol–water formulation (v/v) with four internal standards (0.02 mg/mL L‐2‐chlorophenylalanine, ≥ 98% purity, ADAMAS‐BETA). The mixture underwent homogenization (−10°C, 6 min) followed by ultrasonic‐assisted separation for 30 min at 40 kHz and 5°C. Thereafter, the samples were then precipitated at (−20°C, 30 min) before centrifugation (4°C, 15 min, 13,000 *g*).

UPLC‐MS/MS was employed for the evaluation of the obtained supernatant, using a technique outlined by Shen et al. (2025a, 2025b). The UHPLC‐Triple TOF 6600 system included an ACQUITY UPLC HSS T3 column, with dimensions of 100 mm × 2.1 mm, 1.8 μm (Waters, USA). A water:acetonitrile (95:5, v/v) mixture containing 0.1% formic acid was used as mobile phase A, while acetonitrile:isopropanol:water (47.5:47.5:5, v/v) with 0.1% formic acid was employed for mobile phase B. Other parameters included a 3 μL injection volume and a 40°C chromatographic column temperature, while the gradient elution program consisted of 0%–5% B from 0 to 0.1 min; 5%–25% B from 0.1 to 2 min; 25%–100% B from 2 to 9 min; 100% B from 9 to 13 min; 100%–0% B from 13 to 13.1 min; and 0% B from 13.1 to 16 min to re‐equilibrate the system. A UHPLC‐Q Exactive HF‐X MS system, featuring an electrospray ionization (ESI) source, was employed for analysis in both negative and positive modes. Mass spectrum settings included a capillary temperature of 400°C, an ion‐spray voltage floating (ISVF) of ±3500 V for positive ionization and −3500 V for negative ionization, and standardized fragmentation levels set at 20, 40, and 60 V for MS/MS collection. The mass spectra were acquired between 70 and 1050 m/z. In addition, a pooled quality control sample (QC) was prepared by mixing equal volumes of all samples, which were analyzed in the same manner as the analytic samples.

The pretreatment of raw data was performed by Progenesis QI v3.0 (Waters Corporation, Milford, USA) software. Internal standard peaks and false positive peaks were removed. The compounds were identified by searching databases including HMDB (http://www.hmdb.ca/), Metlin (https://metlin.scripps.edu/), and the Majorbio Database (MJDB). Then, the data matrix obtained was uploaded to the Majorbio cloud platform (https://cloud.majorbio.com) for data analysis. The response intensities of the sample mass spectrometry peaks were normalized using the sum normalization method. Meanwhile, the variables of QC samples with relative standard deviation (RSD) > 30% were excluded and log_10_ logarithmicized.

### 2.5 Sensory Evaluation

The coffee sample sensory assessment, performed by five qualified Q‐grade evaluators (Anke Coffee Co. Ltd., Kunming, China), adhered to the cupping procedure delineated by the Specialty Coffee Association (SCA) (Moon et al. 2025). Ten sensory features, including consistency, sweetness, clean cup, overall impression, balance, body, aftertaste, flavor, fragrance, and aroma, were evaluated on a scale from 6 to 10 with each score in increments of 0.25, which categorized as good (6.00–6.75), very good (7.00–7.75), excellent (8.00–8.75), and outstanding (9.00–10.00). And defects were considered negative scores. Every evaluator assigned a total score of each sample based on the ratings of each attribute under blind conditions, with no prior knowledge of sample identity. Finally, the average score from all evaluators reflected the overall quality of the coffee. Coffee with a final total score less than 80 was regarded as below specialty grade, whereas as specialty grade (80.0–84.99 very good specialty, 85.00–89.99 excellent specialty, and 90.00–100.00 outstanding specialty). Additionally, the body, aftertaste, aroma, and fragrance characteristics and other descriptive sensory attributes were provided based on the Coffee Taster's Flavor Wheel. In addition, the nutty, roasted, flowery, sweet, and fruity coffee aroma values were scored on a 10‐point scale to analysis the characteristics.

### 2.6 Volatile Compound Analysis

Aromatic profiles in roasted coffee matrices were characterized via headspace solid‐phase microextraction (HS‐SPME) in conjunction with gas chromatography–mass spectrometry (GC–MS) (Shen et al. 2025a, 2025b). Sample preparation involved placing 200 mg of finely ground roasted coffee beans in a glass headspace tube (20 mL) and equilibrating the sample for a further 5 min at 60°C. A DVB/CWR/PDMSSPME Arrow fiber (120 μm; Agilent Technologies) was then inserted into the vial headspace and exposed at 60°C for 15 min to absorb volatile compounds. 3‐Heptanone served as the internal standard. The analysis combined a 7000D triple quadrupole mass spectrometer and an Agilent 8890 GC unit, while a DB‐5MS capillary column with dimensions of 30 m × 0.25 mm × 0.25 μm film thickness was employed for separation, applying a helium gas at a constant 1.2 mL/min flow rate and a maintained 250°C injection port temperature. The GC oven temperature program included an initial 3.5 min at 40°C, which was increased at 10°C/min to 100°C. The temperature was then further elevated to 180°C at 7°C/min, and again to 280°C at 25°C/min and held for 5 min. The mass spectra were obtained at 70 eV in electron impact (EI) ionization mode, with 230°C ion source, 280°C transfer line, and 150°C quadrupole mass detector temperatures. The identification of volatile compounds was considered both mass spectrum and retention index (RI) matches. After comparing with the NIST 1.6 and Wiley 6.0 databases, only compounds with similarity > 80% remained for further analysis. While the RI of each volatile compound was calculated using n‐alkanes (C_8_–C_20_) to increase the reliability of the qualitative. RI ± 30 was considered annotated. Isomers with similar spectra were annotated by comparing their RT with that of the measured standards (Yuan et al. 2024).

The following equation was used to calculate the relative odor activity values (rOAV) of the VCs:

$${\text{rOAV}}_{i}=\frac{c_{i}}{T_{i}}$$

where *c*_i_ represents the relative VC concentration, and *T*_i_ denotes the VC odor threshold in water.

### 2.7 Statistical Analysis

The contribution of individual variables was assessed via the variable importance in projection (VIP) scores obtained by the OPLS‐DA model using the R package “ropls” (Version 1.6.2). Compounds exhibiting significant alterations were identified based on the concurrent thresholds of VIP > 1.0, *p* < 0.05, and a fold change (FC) exceeding 1.50 or falling below 0.67. Pearson's correlation analysis was performed to assess the relationship between microorganisms, aroma character, DCn‐VCs, and DCVCs using R Project 3.6.1.

## 3 Results

### 3.1 Microbial Diversity

**FIGURE 1:**
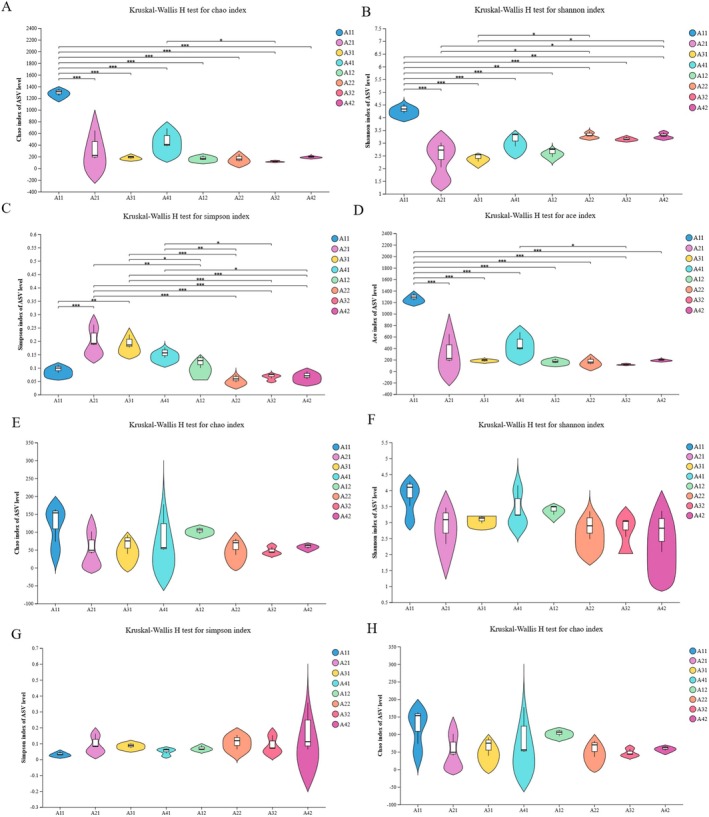
The microbial alpha diversity. (A–D) Chao, Shannon, Simpson, and ACE indices in bacteria, respectively; (E–H) Chao, Shannon, Simpson, and ACE indices in fungi, respectively. “*” indicated a significant difference with *p* < 0.05, “**” indicated an extremely significant with *p* < 0.01, “***” indicated an very extremely significant with *p* < 0.001.

A total of 1,495,609 optimized bacterial sequences and 1,454,771 fungal sequences were obtained, corresponding to 563,771,957 bacterial sequence bases and 454,030,411 fungal sequence bases. The average read lengths were 377 bp for bacterial sequences and 313 bp for fungal sequences. Sequencing coverage ranged from 99.63% to 100% across all samples, ensuring a thorough and dependable characterization of microbial diversity. Alpha diversity analysis (Figure 1) was used to compare microbial biodiversity. The Chao index before fermentation was higher than after fermentation. Meanwhile, Chao, Shannon, and ACE indices showed a trend of first decreasing and then increasing with increasing planting altitude. For bacteria, the Chao, Shannon, and ACE indices before fermentation were higher than after fermentation, reaching the highest values of 1304.02 ± 63.38, 4.35 ± 0.20, and 1295.28 ± 63.60 in A11 and the lowest values of 118.86 ± 10.35 (A32), 2.44 ± 0.18 (A31), and 119.56 ± 11.12 (A32), respectively. The Simpson index ranged from 0.10 ± 0.02 (A11) to 0.22 ± 0.05 (A21) before fermentation, while it ranged from 0.06 ± 0.02 (A22) to 0.12 ± 0.03 (A12) after fermentation. For fungi, A11 also exhibited the highest α‐diversity (Chao, Shannon, and ACE indices), reaching 127.72 ± 54.96, 3.95 ± 0.46, and 106.01 ± 91.94, respectively. The Simpson index ranged from 0.04 ± 0.01 (A11) to 0.11 ± 0.05 (A21) before fermentation, while it ranged from 0.07 ± 0.02 (A12) to 0.18 ± 0.17 (A4) after fermentation.

**FIGURE 2:**
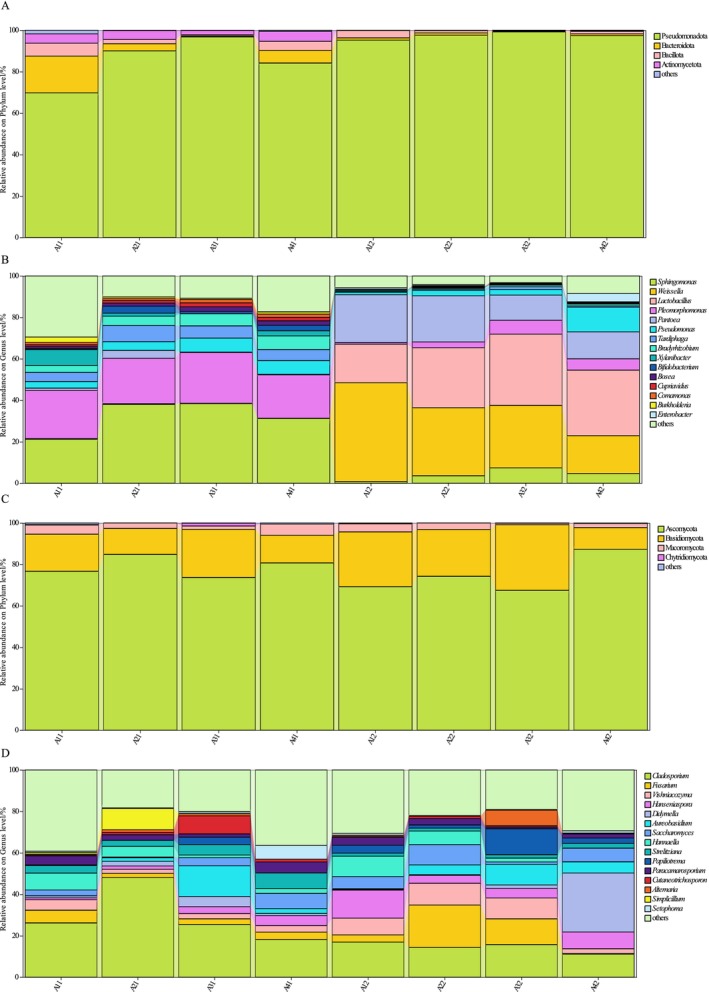
The relative abundance percentage during the wet processing of different planting altitudes, the phylum level of bacteria (A); the genus level of bacteria (B); the phylum level of fungi (C); the genus level of fungi (D).

Five bacterial phyla (Pseudomonadota, Bacteroidota, Bacillota, Actinomycetota, and others) were identified at the phylum level during fermentation (Figure 2A). Pseudomonadota was the dominant bacterial phylum in the wet processing, which increased gradually with fermentation duration, reaching the maximum relative abundance after fermentation. Before fermentation, relative abundance of Pseudomonadota ranged from 69.38% (A11) to 96.82% (A31), while from 95.28% (A12) to 99.26% (A32) after fermentation. Simultaneously, the relative abundance of Pseudomonadota showed a significant change with increasing planting altitude, which reached the maximum relative abundance in A31 and A32. Sixteen genera were identified, including *Sphingomonas*, *Weissella*, *Lactobacillus*, *Pleomorphomonas*, *Pantoea*, *Pseudomonas*, *Tardiphaga*, *Bradyrhizobium*, *Xylanibacter*, *Bifidobacterium*, *Bosea*, *Cupriavidus*, *Comamonas*, *Burkholderia*, *Enterobacter*, and others (Figure 2B). According to the relative abundance on the genus level, *Sphingomonas* (21.25%~38.38%) and *Pleomorphomonas* (20.95%~24.50%) were the dominant bacterial genus before fermentation. Meanwhile, *Sphingomonas* increased with increasing planting altitude, reaching the maximum relative abundance in A31, then decreased to 31.22% in A41. *Pleomorphomonas* also reached 24.50% in A 31. However, *Weissella* (18.27%~47.67%), *Lactobacillus* (18.59%~34.40%) and *Pantoea* (12.17%~23.12%) were the dominant bacterial genus after fermentation. *Weissella* decreased with increasing planting altitude. Meanwhile, *Lactobacillus* increased with increasing planting altitude, reaching the maximum relative abundance in A32, then decreased to 31.63% in A42. On the contrary, *Pantoea* decreased with increasing planting altitude, reaching the minimum relative abundance in A32, then increased to 13.04% in A42.

Linear discriminant analysis effect size (LEfSe) was used to explore the characteristic microorganisms in each planting altitude and select biomarkers. The influence of biomarkers on significantly different groups was evaluated using linear discriminant analysis (LDA) scores. As shown in Figure S1, *Bacteroides*, *Rhizobium*, *Rumiococcus*, *Methylobacterium* were significantly higher in A11, with LDA scores of 4.01, 3.80, 3.64, 3.44, respectively. *Tardiphaga*, *Devosia*, *Bifidobacterium* were substantially higher in A21, with LDA scores of 4.55, 4.20, 4.13, respectively. Ten bacterial genera, such as *Sphingomonas*, *Aquabacterium*, *Rhodopseudomonas* were significantly higher in A31, with LDA scores of 5.27, 4.07, 3.94, respectively. Seven genus‐level bacteria, such as *Bradyrhizobium*, *Delftia*, *Staphylococcus*, *Streptococcus* were significantly higher in A41, with LDA scores of 4.43, 3.76, 3.52, 3.72, respectively. *Weissella* and *Pantoea* were significantly higher in A12, with LDA scores of 5.28 and 4.96, respectively. *Gammaproteobacteria* and *Enterococcus* were substantially higher in A22, with LDA scores of 4.61 and 3.79, respectively. *Lactobacillus* was significantly higher in A32 with LDA scores of 5.13. *Pseudomonas*, *Erwiniaceae*, *Chryseobacterium*, and *Massilia* were significantly higher in A42, with LDA scores of 4.64, 3.91, 3.89, 3.76, respectively.

In fungi communities, five phyla were identified: Ascomycota, Basidiomycota, Mucoromycota, Chytridiomycota, and others (Figure 2C). *Ascomycota* was the dominant fungal phylum, which ranged from 73.71% to 84.82% before fermentation, and from 67.48% to 87.33% after fermentation. The maximum relative abundance was 84.82% in A21 and 87.33% in A42, respectively. After *Ascomycota*, *Basidiomycota* was also an important fungal phylum. Sixteen genera were identified, including *Cladosporium*, *Fusarium*, *Vishniacozyma*, *Hanseniaspora*, *Didymella*, *Aureobasidium*, *Saccharomyces*, *Hannaella, Strelitziana*, *Papiliotrema*, *Paracamarosporom*, *Cutaneotrichosporon*, *Alternaria*, *Simplicillium*, *Setophoma*, and others, which showed significant diversity (Figure 2D). Based on the relative prevalence of genus rank, *Cladosporium* was the dominant fungal genus, which significantly decreased with fermentation duration, ranging from 18.14% (A41) to 48.06% (A21) before fermentation, from 11.13% (A42) to 16.94% (A12) after fermentation. *Fusarium* was also the main fungal genus in A22 (20.50%) and A32 (12.44%). Moreover, five genus level fungi including *Cyphellophora*, *Hanseniaspora*, *Hannaella*, *Papiliotrema* and *Didymella* were significantly higher in four group, with LDA scores of 4.58 (A11), 4.79 (A12), 4.78 (A12), 4.90 (A32), 5.14 (A42), respectively.

### 3.2 Non‐Volatile Compounds Results

UPLC‐MS/MS analysis identified 2290 non‐volatile compounds (n‐VCs) during the wet processing of coffee beans across various altitudes, encompassing 16 super‐classes. 554 lipids and lipid‐like molecules; 461 organic acids and derivatives; 310 organoheterocyclic compounds; 299 organic oxygen compounds; 273 phenylpropanoids and polyketides; 229 benzenoids; 54 nucleosides, nucleotides, and analogs; 28 organic nitrogen compounds; 23 lignans, neolignans, and related compounds; 17 alkaloids and derivatives; seven hydrocarbons; two organosulfur compounds; two allenes; two hydrocarbon derivatives; one homogeneous non‐metal compound; and 28 not available compounds were identified during the wet processing of coffee from different planting altitudes, as shown in Figure S2.

**FIGURE 3:**
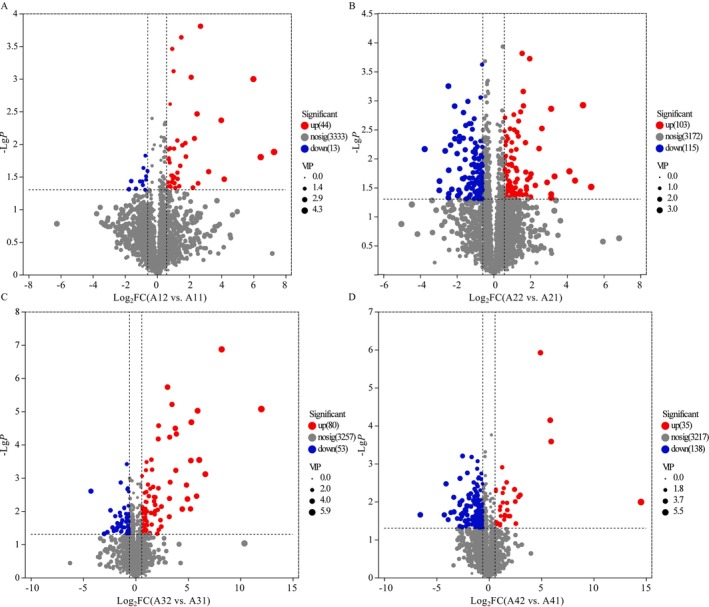
The differentially changed non‐volatile compounds (DCn‐VCs) between A12 vs. A11, A22 vs. A21, A32 vs. A31, A42 vs. A41. A total of 57 DCn‐VCs were found between A12 and A11 (A), including 44 up‐regulated and 13 down‐regulated DCn‐VCs; 218 DCn‐VCs were identified between A22 and A21 (B), including 103 up‐regulated and 115 down‐regulated DCn‐VCs; 133 DCn‐VCs were found between A32 and A31 (C), including 80 up‐regulated and 53 down‐regulated DCn‐VCs; and 173 DCn‐VCs were identified between A42 and A41 (D), including 35 up‐regulated and 138 down‐regulated DCn‐VCs.

Differentially changed non‐volatile compounds (DCn‐VCs) arising from the wet processing of coffee cultivated at varying altitudes were identified according to the parameters of VIP higher than 1.0, *p* < 0.05, and an FC over 1.5 or lower than 0.67, which were shown in Table 1. These DCn‐VCs compounds in the comparison between after fermentation and before fermentation were shown in Figure 3. In the A12 versus A11 comparison (Figure 3A), the up‐regulated DCn‐VCs included valproic acid, phloionolic acid, galacturonic acid, isopentenyladenine‐9‐N‐glucoside, protocatechuic acid, hydroquinone, 2‐O‐methylascorbic acid, 6‐dehydrotestosterone glucuronide, 5‐dehydroquinic acid, exotoxin, tuberosin, and N‐arachidonoyl asparagine, etc. Conversely, the down‐regulated DCn‐VCs included 6‐gingerol, capsoside A, musabalbisiane A, 3′‐glucosyl‐2′,4′,6′‐trihydroxyacetophenone, pectolinarin, sterigmatocystin, etc.

**TABLE 1:** The differentially changed non‐volatile compounds (DCn‐VCs) in coffee fermentation.

DCn‐VCs	A12 vs. A11		A22 vs. A21		A32 vs. A31		A42 vs. A41		A42 vs. A12		A42 vs. A22		A42 vs. A32	
	Up	Down	Up	Down	Up	Down	Up	Down	Up	Down	Up	Down	Up	Down
Lipids and lipid‐like molecules	18	2	25	42	31	13	11	49	5	32	8	18	7	25
Organic oxygen compounds	6	2	14	16	6	4	4	14	9	4	7	4	8	11
Benzenoids	5	5	3	3	9	1	1	9	8	9	2	4	5	6
Organoheterocyclic compounds	5	2	15	14	11	14	5	17	5	8	5	12	5	14
Organic acid and derivatives	4	0	22	16	10	10	6	23	4	6	11	7	11	12
Lignans, neolignans and related compounds	2	0	0	5	0	2	0	5	2	2	0	1	2	3
Nucleosides, nucleotides, and analogues	2	0	4	4	0	2	1	3	1	3	1	1	0	1
Phenylpropanoids and polyketides	1	2	16	11	11	6	4	12	7	4	4	3	6	3
Organic nitrogen compounds	0	0	1	1	1	0	0	1	0	0	0	0	0	0
Alkaloids and derivatives	0	0	2	1	1	0	0	2	0	0	0	0	0	2
Hydrocarbon derivatives	0	0	0	0	0	0	0	1	0	0	0	0	0	1
Not available compounds	1	0	1	2	0	1	3	2	1	1	2	1	1	0
Total	44	13	103	115	80	53	35	138	42	69	40	51	45	78

Similarly, 103 up‐regulated DCn‐VCs (e.g., 5‐dehydroquinic acid, N‐carboxyacetyl‐D‐phenylalanine, spinosin, 2‐*O*‐methylascorbic acid, canescein, linusitamarin, panthenol, 6‐gingerol, anacardic acid, argentine, dihydroisomorphine‐6‐glucuronide, etc.) and 115 down‐regulated DCn‐VCs (e.g., 2‐polyprenyl‐6‐methoxyphenol, gluconolactone, neohesperidoside, 2′‐(E)‐feruloyl‐3‐(arabinosylxylose), isoorientin 6‐*O*‐Hexoside, adenylo‐succinate, 5′‐S‐methyl‐5′‐thioadenosine, M‐cresol, alpha‐linoleoylcholine, N‐arachidonoyl asparagine, etc.) were detected between A22 and A21 (Figure 3B).

In the comparison between A32 and A31 (Figure 3C), 80 up‐regulated DCn‐VCs included 3‐hydroxy‐4‐methoxyphenyllactic acid, pinostilbenoside, nicotyrine, 2‐*O*‐methylascorbic acid, betalamic acid, lactupicrin, leonuriside A, 1‐phenyl‐1,3‐dodecanedione, ergovaline, convalloside, etc. And 53 down‐regulated DCn‐VCs included 2‐(4‐hydroxyphenylazo)benzoic acid, lycoperdic acid, *L*‐pyridosine, *p*‐coumaroyl glycolic acid, lusitanicoside, cellobiose, hesperetin 7‐neohesperidoside, 5′‐S‐methyl‐5′‐thioadenosine, deoxyinosine, N‐arachidonoyl asparagine, etc.

In the comparison between A42 and A41 (Figure 3D), 35 up‐regulated (e.g., phloionolic acid, 5‐dehydroquinic acid, *N*‐nervonoyl methionine, 2‐*O*‐methylascorbic acid, 2′,3′‐didehydro‐2′,3′‐dideoxyguanosine, malvidin 3‐Alpha‐L‐galactoside, clozapine glucuronide, hydroquinone, 5′‐*O*‐methylthymidine, etc.) and 138 down‐regulated DCn‐VCs (e.g., atractyligenin 2‐glucuronide, glycyl‐phenylalanine, 3‐hydroxydodecanedioic acid, lusitanicoside, 4‐hydroxybenzyl alcohol, citrusin B, myricatomentoside I, 5′‐S‐methyl‐5′‐thioadenosine, dihydrocytochalasin B, N‐arachidonoyl asparagine, etc.) were found.

**FIGURE 4:**
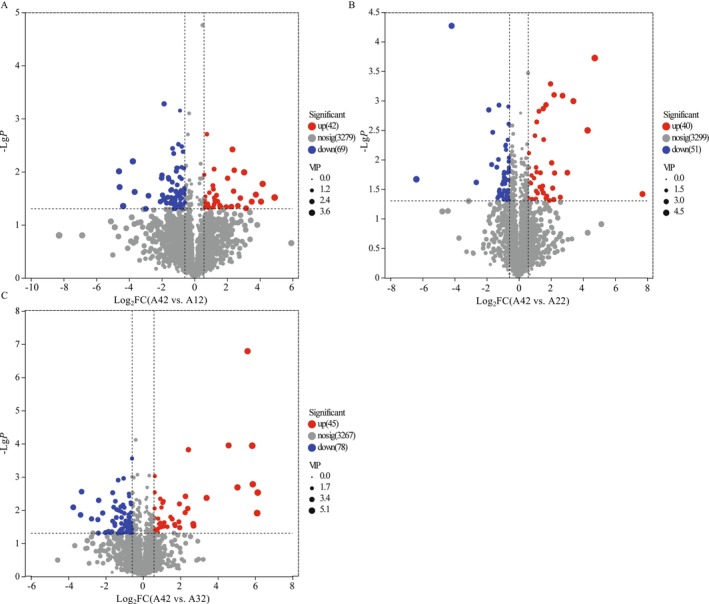
The differentially changed non‐volatile compounds (DCn‐VCs) between A42 vs. A12, A42 vs. A22, and A42 vs. A32. A total of 111 DCn‐VCs were found between A42 and A12 (A), including 42 up‐regulated and 69 down‐regulated DCn‐VCs; 91 DCn‐VCs were identified between A42 and A22 (B), including 40 up‐regulated and 51 down‐regulated DCn‐VCs; and 123 DCn‐VCs were found between A42 and A32 (C), including 45 up‐regulated and 78 down‐regulated DCn‐VCs.

The DCn‐VCs were also compared between high planting altitude A4 and low planting altitude, as shown in Figure 4. In the comparison between A42 (high altitude) and A12 (lower altitude) (Figure 4A), a total of 111 DCn‐VCs were identified, including 42 up‐regulated (e. g., tuliposide B, panthenol, propargite, pinostilbenoside, 4′,8‐dimethylgossypetin 3‐glucoside, (3*β*,20R,22R)‐3,20,27‐trihydroxy‐1‐oxowitha‐5,24‐dienolide 3‐glucoside, hypochoeroside A, 6‐oxopiperidine‐2‐carboxylic acid, (‐)‐2‐difluoromethylornithine, matairesinoside and hesperetin 7‐neohesperidoside, etc.) and 69 down‐regulated DCn‐VCs (e.g., 15‐hydroxynorandrostene‐3,17‐dione glucuronide, protocatechuic acid, hydroquinone, 6‐dehydrotestosterone glucuronide, borapetoside, 3‐hydroxydodecanedioic acid, 2‐guanidinobutanoic acid, isopentenyladenine‐9‐N‐glucoside, sexangularetin 3‐glucoside 7‐rhamnoside, 4′‐O‐methyl‐epicatechin glucuronide, exotoxin, N‐arachidonoyl asparagine, etc.).

In the comparison between A42 and A22 (Figure 4B), 91 DCn‐VCs emerged, with 40 up‐regulated (e.g., 5‐dehydroquinic acid, 6‐oxopiperidine‐2‐carboxylic acid, cis‐linoleic acid, neohesperidoside, stachyose, 1‐*O*‐caffeoylglucose, 1‐naphthol, N‐phenylanthranilic acid, *p*‐nitrophenyl thymidine 5′‐monophosphate, etc.) and 51 down‐regulated DCn‐VCs (e.g., plantamajoside, 6,7‐octadienoylglycine, lysyl‐phenylalanine, 5‐hexenyl glucosinolate, 2‐phenylethyl Beta‐D‐glucopyranoside, heraclenol, clusin, indole carboxylic acid sulfate) were identified.

A42 and A32 (Figure 4C) were compared, resulting in the identification of 123 DCn‐VCs. The 45 up‐regulated DCn‐VCs (e.g., N‐acetylgalactosaminyl lactose, salicylic acid, 2‐amino‐2‐deoxy‐D‐gluconic acid, 8‐deoxylactucin, hesperetin 7‐neoheperidoside, 1‐naphthol, salicylic acid, epicatechin, p‐coumaroyl glycolic acid, 5‐phenylvaleric acid, myrianthic acid, etc.) and 78 down‐regulated DCn‐VCs (e.g., caffeic acid, cinnamic acid, pretyrosine, 4‐hydroxybenzyl alcohol, 2‐ethyl‐4‐(2‐furanyl)‐2‐propenal, atractyligenin 2‐glucuronide, valproic acid, linusitamarin, 1,5‐anhydroglucitol, *β*‐thujaplicin, citrusin B, clusin, dihydroisomorphine‐6‐glucuronide and ergovaline, etc.).

### 3.3 Sensory Characteristics

Each sample received a top score of 10 in sugary note, purity, and consistency, while other cupping attributes ranged between 6.75 and 7.75 (Figure S3A). The total scores of different planting altitudes were 78.17 (A1), 79.33 (A2), 80.08 (A3), and 82.75 (A4), respectively. Roasted coffee beans above 1200 m were classified as very good. The cupping score increased with increasing planting altitude and the coffee growing in high altitudes tends to obtain specialty coffee according to the SCA with above 80 points in sensory tests (Oliva‐Cruz et al. 2024; Moon et al. 2025). A4 exhibited a long‐lasting aftertaste, mellow, and full body. Furthermore, the aroma score results (Figure S3B) showed that A4 exhibited higher sweet, nutty, and flowery scores than others. This indicated that high altitude is a very important factor influencing coffee flavor quality.

### 3.4 HS‐SPME‐GC–MS Results

**FIGURE 5:**
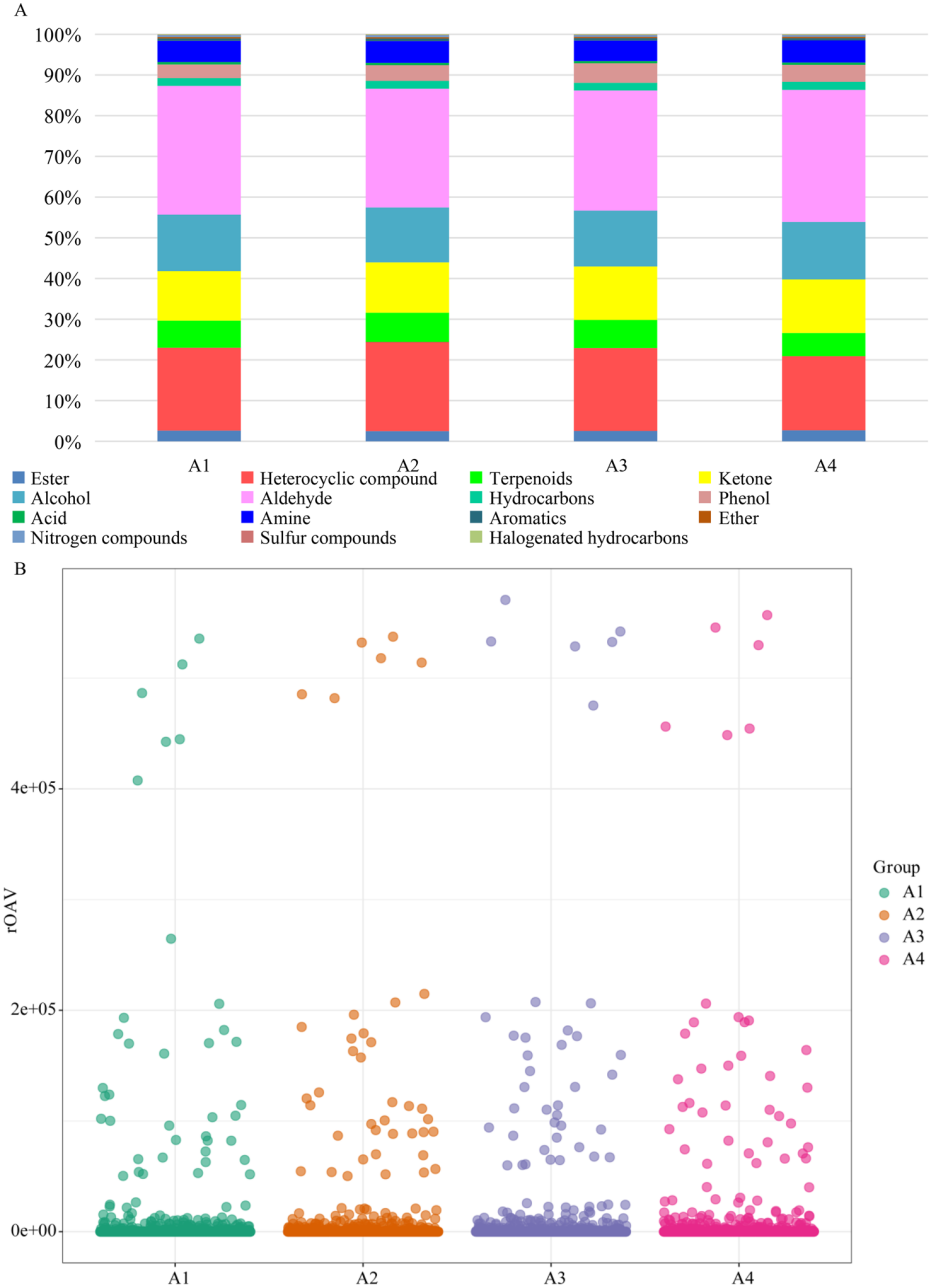
The results of volatile compounds analysis. The classification of volatile compounds (A); rOAV from different planting altitudes coffee (B).

To further elucidate the influence of planting altitude on coffee flavor, the volatile compound (VC) profiles of roasted coffee samples were analyzed using HS‐SPME‐GC–MS. A total of 15 classes including 1234 VCs were detected, which included 194 esters, 163 heterocyclic compounds, 159 terpenoids, 148 ketones, 146 alcohols, 98 aldehydes, 67 hydrocarbons, 50 phenols, 46 acids, 45 amines, 37 aromatics, 37 ethers, 28 nitrogen compounds, 9 sulfur compounds, and 7 halogenated hydrocarbons. The relative contents of these classes were shown in Figure 5A. Among them, 651 VCs (126 esters, 103 heterocyclic compounds, 101 terpenoids, 84 ketones, 73 aldehydes, 66 alcohols, 23 ethers, 23 phenols, 17 acids, 11 aromatics, 8 hydrocarbons, 7 sulfur compounds, 6 amines, and 3 nitrogen compounds) exhibited rich aroma such as sweet (e.g., (E)‐cinnamaldehyde; ethanone, 1‐(2,4‐dimethylphenyl)‐; 2‐acetyl‐5‐methylthiophene; etc.), fruity (e.g., ethyl tiglate; 3,5‐octadien‐2‐one; 2H‐pyran‐2‐one, tetrahydro‐; etc.), flowery (e.g., trans‐geranic acid methyl ester; anethole; lilac aldehyde D; etc.), roasted (e.g., furan, 2,5‐dimethyl‐; p‐mentha‐1,5,8‐triene; phenol, 2,4‐dimethyl‐; etc.), caramel (e.g., furaneol; isomaltol; 2‐butenoic acid, hexyl ester; etc.), nutty (e.g., thiazole, 5‐ethyl‐2,4‐dimethyl‐; phenol, 2‐methoxy‐; ethanone, 1‐(4,5‐dihydro‐2‐thiazolyl)‐; etc.), cocoa (e.g., Benzeneacetaldehyde, alpha‐(2‐methylpropylidene)‐; pyrazine, methyl‐; furan, 2‐ethyl‐; etc.), honey aromas (e.g., 2‐acetyl‐5‐methylthiophene, phenethyl acetate, (E)‐β‐damascone), chocolate (e.g., thiazole, 5‐ethyl‐2‐methyl‐; pyrazine, methyl‐; acetylpyrazine; etc.), milky (e.g., 4‐heptenal, (Z)‐; 3‐(Methylthio)‐2‐butanone; Hexadecanoic acid, ethyl ester; etc.), cherry, pineapple, lemon, apple, banana, plum, hyacinth and other aroma characteristics. Simultaneously, according to the rOAV (Figure 5B) 362 VCs (59 heterocyclic compounds, 57 esters, 49 alcohols, 48 aldehyde, 46 ketones, 38 terpenoids, 26 phenols, 15 aromatics, 5 nitrogen compounds, 5 ethers, 5 acids, 4 amines, 2 sulfur compounds, 2 halogenated hydrocarbons, and 1 hydrocarbon) contributed directly to the flavor of the coffee with an rOAV over 1.0. Among them, 1‐nonen‐3‐one; 2‐furfurylthiol; 3(2H)‐furanone, dihydro‐2‐methyl‐; pentanoic acid, 2‐methyl‐, ethyl ester; pyrazine, 2‐methoxy‐3‐(2‐methylpropyl)‐; ethyl 3‐cyclohexenecarboxylate; ethanone, 1‐(2‐aminophenyl)‐; pyrazine, 2‐methoxy‐3‐(1‐methylpropyl)‐; pyrazine, 2‐ethyl‐3,5‐dimethyl‐; pyrazine, 2,3‐diethyl‐5‐methyl‐; and 6‐nonenal, (E)‐were identified as the most critical VCs due to their maximal rOAV values and essential contributions to coffee flavor.

The DCVCs with FC < 0.67 or FC > 1.50, VIP > 1.0, and *p* < 0.05 among different planting altitudes were assessed and identified to gain insight into the change of the planting altitude (Table S1). 42 DCVCs were found between A4 versus A1, including 26 higher and 16 lower DCVOCs. 27 DCVCs were identified in A4 versus A2, of which nine were decreased and 18 increased. 26 DCVCs were detected in A4 versus A3, including 12 up‐regulated and 14 down‐regulated DCVCs. These DCVCs contributed to coffee aroma characteristics, shown in Figure S4. For example, among them, 7 DCVCs (Methyl isovalerate; propanoic acid, 2‐methyl‐, butyl ester; 2‐hydroxy‐3‐methyl‐, methyl ester; pentanoic acid, hexadecanoic acid, ethyl ester; 1,3,7‐octatriene, 3,7‐dimethyl‐; 4‐pyridinecarboxaldehyde; and pentanoic acid, 2‐hydroxy‐4‐methyl‐, methyl ester) correlated with fruity aromas. 10 DCVCs (propanoic acid, 2‐methyl‐, butyl ester; trans‐beta‐Ocimene; decanal; 3‐(methylthio)propanoic acid methyl ester; 3‐octanone; benzene, 1,2,4,5‐tetramethyl‐; 2,4,6‐octatriene, 2,6‐dimethyl‐; and 2,4,6‐octatriene, 2,6‐dimethyl‐, (E,E)‐ pentanoic acid, 2‐hydroxy‐4‐methyl‐, methyl ester) contributed to sweet aromas. Additionally, 2‐furancarboxylic acid and methyl 2‐hydroxy‐3‐methylpentanoate were related to caramel aroma. Compounds such as benzene, (2‐methoxyethyl)‐; 1,3,7‐octatriene, 3,7‐dimethyl‐; decanal; (E,E)‐2,6‐dimethyl‐2,4,6‐octatriene and trans‐rose oxide; and 2‐nonynoic acid, methyl ester were associated with floral aroma characteristics.

**FIGURE 6:**
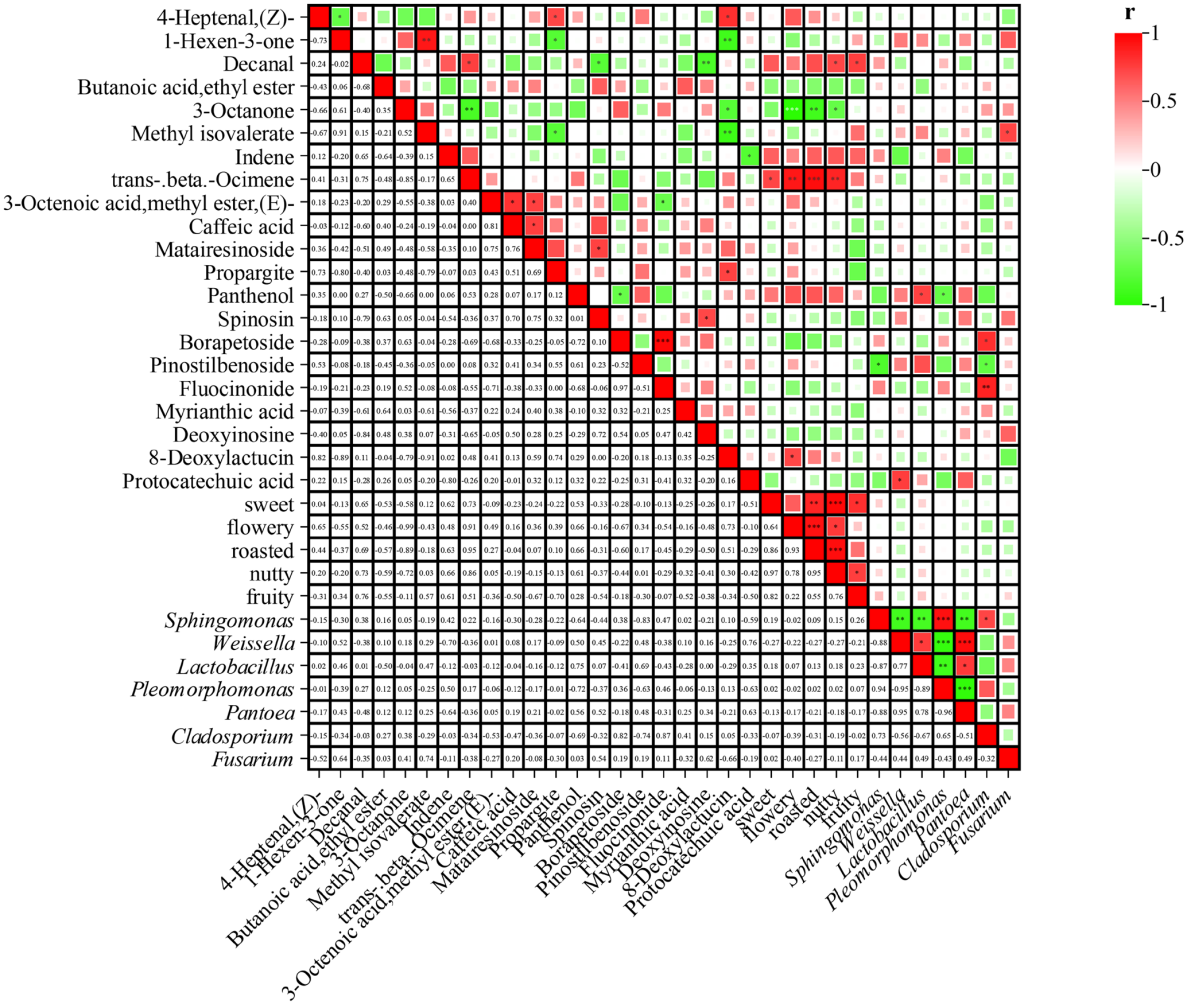
The relationship between microorganisms, aroma character, DCn‐VCs and DCVCs. *p < 0.05, **p < 0.01, ***p < 0.001.

To better understand and demonstrate the function of microbiota on coffee flavor, Figure 6 showed the relationships among microorganisms, aroma character, DCn‐VCs, and DCVCs employing Pearson's correlation analysis. Based on the value of the correlation coefficient (*r*), 0.80–1.0 indicated an extremely strong correlation (Hu et al. 2020). Methyl isovalerate showed an extremely strong positive correlation with 1‐hexen‐3‐one with the value of *r* 0.91. Trans‐beta‐Ocimene showed an extremely strong negative correlation with 3‐Octanone (*r* = −0.85). Caffeic acid was an extremely strong positive correlation with 3‐Octenoic acid, methyl ester, (E)‐ (*r* = 0.81). Fluocinonide was an extremely strong positive correlation with borapetoside (*r* = 0.97). 8‐deoxylacyucin was extremely strong positive correlation with 4‐heptenal, (Z)‐ (*r* = 0.82), and extremely strong negative correlation with 1‐hexen‐3‐one (*r* = −0.89) and methyl isovalerate (*r* = −0.91), respectively. Protocatechuic acid showed an extremely strong negative correlation with indene (*r* = −0.80). Moreover, the correlation analysis between sensory flavor and chemical compounds showed that coffee flowery was extremely strong negative correlation with 3‐Octanone (*r* = −0.99). Trans‐beta‐Ocimene was extremely strong positive correlation with coffee flowery (*r* = 0.91), roasted (*r* = 0.95), and nutty (*r* = 0.86). *Cladosprium* was extremely strong positive correlation with fluocinonide (*r* = 0.87).

## 4 Discussion

The coffee flavor is the result of the interaction of various flavor compounds in coffee, such as caffeine, chlorogenic acid, trigonelline, phenols, carboxylic acids, polysaccharides, lipids, alcohols, ketones, esters, furans, and aldehydes (Sunarharum et al. 2014; Pua et al. 2022). For instance, caffeine, trigonelline, lipids, sugars and chlorogenic acid contribute to the flavor, aroma or bitter taste attributes of coffee (Cassamo et al. 2022; Torrez et al. 2023). However, the formation and composition of these flavor compounds are influenced by a multitude of factors, including coffee species, geographical origin, climatic conditions, cultivation practices, post‐harvest primary processing methods, storage conditions, degree of roasting, organoleptic qualities, and other vital factors (He et al. 2025; Zhai et al. 2024). Under the interaction of these factors, the formation and accumulation of coffee flavor precursor substances such as lipids, polysaccharides, sucrose, proteins, chlorogenic acid, caffeine, and trigonelline, undergo differential accumulation in green coffee beans. During roasting, these precursors participate in Maillard reactions, Strecker degradation, caramelization, and thermal fragmentation to produce aroma‐active compounds (Lee et al. 2015). A multiple‐factor comprehensive analysis based on different continents, altitudes, and post‐harvest processing methods showed that altitude contributed 74.2% to coffee flavor characteristics according to the score of the Random Forest model (Kim et al. 2025). As an important factor influencing coffee quality, the planting altitude of coffee can enhance the aroma and flavor with increasing altitudes and affect the chemical composition of coffee.

In wet processing, microbiomes have a significant impact on the fermentation and quality of coffee. This method introduces a diverse microbial community, comprising bacteria, yeasts, and filamentous fungi sourced from the environment, equipment, human contact, and other physical contacts to degrade coffee mucilage and remove coffee layers (Elhalis et al. 2023; Shen et al. 2023; Ferreira et al. 2023). Microbial richness and dominant microorganism in fermentation showed significant differences with different planting altitudes (Martinez et al. 2022). The Alpha diversity (Chao, Shannon, and ACE index) was employed to reflect microbial community richness, microbial community diversity (Liu et al. 2025; Wang et al. 2023). The Chao index suggested that the low‐planting altitude (A1) coffee presented higher microbial richness. The Shannon index suggested that microbial community diversity in the low‐planting altitude (A1) exhibited greater complexity. *Sphingomonas* in high‐planting altitude was higher than in the low‐planting altitude (A1). For fungi, *Cladosporium*, the dominant fungal genu, significantly decreased with altitude. Moreover, baesd on the LefSe analysis, an efficient way to select biomarkers, identified pivotal genus in different planting altitudes during the wet fermentation (Chang et al. 2022).

An important study showed that planting altitude shapes the dynamics of microbial communities, which further influence microbial biomass and moisture by the abundance and richness of soil microorganisms (Tapaça et al. 2024). This study also showed different metabolic adaptations by different altitudes. In addition, the influence of planting altitude on microbial composition is closely linked to temperature, oxygen, and other climatic factors relating to planting altitude (Costa et al. 2024). Therefore, coffee from different planting altitudes provides rich and diverse microorganisms in the wet processing. Moreover, Martinez et al. (2022) found the most abundant bacterial genera associated with pulped coffee fruits under self‐induced anaerobic fermentation were *Gluconobacter* (800 m), *Weissella* (1000 m), and *Leclercia* (1200 and 1400 m). *Methylobacterium*, *Mangrovibacter*, *Azotobacter* (at 800 m), *Ochrobactrum* (at 1000 m), *Rosenbergiella*, *Yersinia* (at 1200 m), and *Curtobacterium* (at 1400 m) were distinctive at each altitude. *Cystofilobasidium infirmominiatum* was the most abundant fungal genus. Veloso et al. (2020) found an increase in bacterial diversity on the natural microbiota of coffee cherries with increasing altitude, while fungal richness decreased. Therefore, the coffee microbial community structure at different planting altitudes must be considered in the post‐harvest processing methods.

Simultaneously, during coffee fermentation, two primary mechanisms can alter the bean's chemical composition (direct modulation of the endogenous metabolic activity of coffee seeds and migration of microbial metabolites into the bean). These processes contribute significantly to variations in coffee flavor and cupping quality (Elhalis et al. 2023). Planting altitude influences the chemical composition of coffee fruits and beans both before and after harvesting (Martinez et al. 2022). For instance, a previous study reported that lipids, trigonelline, citrate, and malate were key discriminant compounds in coffees grown at altitudes below 969 m, whereas quinic acid, caffeine, and formic acid predominated in samples from altitudes above 1000 m (Oliveira et al. 2022). Therefore, planting altitude changes the coffee fruits and beans composition before and after harvesting. According to the analysis results of non‐volatile compounds using UPLC‐MS/MS, some significant differences in compounds were found. UPLC‐MS/MS is characterized by high separation efficiency and enables effective separation and quantification. UPLC‐MS/MS employs advanced column packing materials and optimized operational parameters to improve separation performance, narrower peak widths, reduced co‐elution interference, enhanced analytical sensitivity, and increased overall efficiency (Zhang, Yan, et al. 2025; Zhang, Zhou, et al. 2025). These DCn‐VCs were the results of combined effect of different microbial fermentations and endogenous chemical compounds from different planting altitudes. Therefore, the chemical compositions from different planting altitudes coffee are affected by endogenous chemical compounds of coffee seeds and microbial fermentation originating from different altitudes.

The Specialty Coffee Association (SCA) framework defines what qualifies as specialty coffee for the international market and coffee researchers, which provides a standardized scoring system to keep a fair and attractive coffee trade (Moon et al. 2025; CarolinaVieira‐Porto et al. 2024). High‐planting altitude can obtain a beverage with an unusual sensory profile (Martins et al. 2020). Coffee flavor improved with increasing planting altitude, as determined by the SCA evaluation. Simultaneously, roasted coffee beans above 1200 m were classified as very good. High‐planting altitude (A4) tends to have a stronger sweet, nutty, and flowery attribute than low‐planting altitude. Therefore, the adjusting way and mechanism of high altitude for coffee flavor is worth further study. Correlation analysis between coffee sensory evaluation and coffee chemical compounds revealed that toluene, benzaldehyde, cresol, citric acid, and benzoic acid were positively correlated with overall, whereas gluconic acid showed a negative correlation. Citric acid, benzoic acid, and ferulic acid were negatively correlated with balance. L‐aspartic acid exhibited a negative correlation with fragrance/aroma; however, chlorogenic acid was a positive correlation with fragrance/aroma. A positive correlation was detected between caffeic acid and acidity (Zhu et al. 2025).

Aroma compounds act as key contributors to the sensory quality and market value of coffee. To clear the influence of planting altitude on coffee flavor compounds, HS‐SPME‐GC–MS, an effective identifying technology on key aroma components in the food field, was employed to analyze volatiles (Wang, Liu, et al. 2025; Wang, Quan, et al. 2025; Li et al. 2024). HS‐SPME‐GC–MS technique is widely employed to qualitative and quantitative analysis of aroma substances in food flavor studies for its high accuracy, high sensitivity, and excellent separation capability (Li et al. 2025; Zhang, Yan, et al. 2025; Zhang, Zhou, et al. 2025). Notably, furanones, phenolics, organosulfur constituents, and pyrazines are key aromatic volatiles that contribute significantly to coffee's sensory profile (Sunarharum et al. 2014). This study identified 651 volatile constituents exhibiting aromatic characteristics, as well as 362 VCs that contribute directly to the flavor of the coffee, among a total of 1234 VCs. In addition, 42, 27 and 26 DCVCs were detected in, respective comparisons of A4 with A1, A2, and A3. These DCVCs are essential contributors to the altitudinal variation in coffee flavor. Moreover, compounds such as methyl isovalerate, propanoic acid, 2‐methyl‐, butyl ester, pentanoic acid, 2‐hydroxy‐4‐methyl‐, methyl ester, 1,3,7‐octatriene, pentanoic acid, 2‐hydroxy‐3‐methyl‐, methyl ester, hexadecanoic acid, ethyl ester, and 4‐pyridinecarboxaldehyde, 2,4,6‐octatriene, 2,6‐dimethyl‐, trans‐beta‐ocimene, pentanoic acid, 2‐hydroxy‐4‐methyl‐, methyl ester, 3‐octanone, δ‐nonalactone, 3‐(methylthio)propanoic acid methyl ester, decanal, benzene, 1,2,4,5‐tetramethyl‐, 2‐furancarboxylic acid, benzene, (2‐methoxyethyl)‐; 1,3,7‐octatriene, 3,7‐dimethyl‐, decanal, trans‐rose oxide, and 2‐nonynoic acid, methyl ester and other DCVCs were related to fruity, sweet, carame, floral aroma of coffee, which maybe closely linked to the planting altitude.

In summary, coffee is significantly influenced by planting altitude by changing its bacterial microorganisms and chemical compounds. And microorganisms originally from different planting altitudes provide a rich starter in the wet processing in the future.

## 5 Conclusions

In common, coffee flavor involves each step of production from farm to cup. This research examined shifts in microbial diversity along with variations in non‐volatile and volatile compound profiles in coffees cultivated at different altitudes. Compared with before fermentation (*Sphingomonas* and *Pleomorphomonas*), the most abundant genera changed to *Weissella* and *Lactobacillu* after fermentation. Meanwhile, the non‐volatile compounds also significantly changed with the planting altitude and fermentation duration. The cupping score increased with increasing planting altitude, and the coffee growing in high altitudes exhibited a long‐lasting aftertaste, mellow and full body with high sweet, nutty, and flowery scores. Furthermore, the differentially changed volatile compounds among different planting altitudes were found between different planting altitudes. Some of them contributed to flavor characteristics for different planting altitudes coffee. Therefore, coffee flavor is significantly influenced by planting altitude through changing microbial diversity and chemical compounds. In addition, microorganisms from different altitudes will provide a potential starter in the coffee fermentation for beneficial coffee flavor.

## Author Contributions

**Bintao Peng:** software, resources. **Kunyi Liu:** methodology, writing – review and editing, project administration. **Wenhua Chen:** resources, software. **Xiaojing Shen:** data curation, writing – original draft, methodology. **Ying Yang:** software, resources. **Surui Lu:** resources, software. **Qi Wang:** data curation, formal analysis, writing – review and editing. **Jilai Zhang:** methodology, writing – review and editing. **Yamei Wu:** methodology, data curation, writing – original draft.

## Funding

This work was supported by the Yunnan Fundamental Research Projects‐Yunnan Province Agricultural Basic Research Joint Foundation (No. 202501BD070001‐004); Yunnan Province Reserve Talent Project for Young and Middle‐Aged Academic and Technical Leaders (No. 202405AC350064); Talent Cultivation Project in Yunnan Province (No. XDYC‐QNRC‐2022‐0039); Yunnan Province Major Science and Technology Project (No. 202402AE090015); Lower Nu River, Mountain Agroecosystem, Observation and Research Station of Yunnan Province (No. 202305AM340031); the Innovation and Entrepreneurship Project for University Students in Yunnan and Sichuan Province (No. S202410676038 and S202512966070); Scientific Research Project of Yibin Vocational and Technical College (No. 24JBGS‐05).

## Disclosure

Author Approval and Responsibility Statement: All authors have read and approved the final version of the manuscript. The corresponding author had full access to all of the data in this study and takes complete responsibility for the integrity of the data and the accuracy of the data analysis.

## Ethics Statement

Ethical approval was not necessary for this sensory evaluation in this research. Because coffee is a safe beverage worldwide beverages and the coffee was processed using a conventional common method. In addition, the certified experts from Anke Coffee Limited Company (Kunming, China) voluntarily participated in the sensory assessment and agreed to the publication of the findings. Furthermore, their rights and privacy of all the participants were protected through proper procedures.

## Conflicts of Interest

The authors declare no conflicts of interest.

## Supporting information


**Table S1:** DCVCs between different planting altitudes.
**Figure S1:** Linear discriminant analysis of bacterial and fungal communities in samples.
**Figure S2:** Super‐classes of chemical compounds during the coffee fermentation of different planting altitudes, the different colors represented different super‐classes, while the numbers indicated the number of the class within each super‐class.
**Figure S3:** Coffee flavor characteristics of different planting altitudes, scores from the coffee cupping test (A), aroma score (B).
**Figure S4:** The flavor characteristics of DCVCs in A4 vs. A1 (A); the flavor characteristics of DCVCs in A4 vs. A2 (B); the flavor characteristics of DCVCs in A4 vs. A3 (C).

## Data Availability

The data that support the findings of this study are available on request from the corresponding author. The data are not publicly available due to privacy or ethical restrictions.
