# Supplementary material for: Interaction Among Microbiota, Chemical Composition, and Quality Variations in Coffea arabica Across Altitudinal Gradients During Wet Processing
Source: Food Sci Nutr. 2026 Jul 30;14(8):e72145. doi: 10.1002/fsn3.72145 (PMC13425609; doi:10.1002/fsn3.72145)
Supplement: Supplementary file 1 — Table S1: DCVCs between different planting altitudes. Figure S1: Linear discriminant analysis of bacterial and fungal communities in samples. Figure S2: Super‐classes of chemical compounds during the coffee fermentation of different planting altitudes, the different colors represented different super‐classes, while the numbers indicated the number of the class within each super‐class. Figure S3: Coffee flavor characteristics of different planting altitudes, scores from the coffee cupping test (A), aroma score (B). Figure S4: The flavor characteristics of DCVCs in A4 vs. A1 (A); the flavor characteristics of DCVCs in A4 vs. A2 (B); the flavor characteristics of DCVCs in A4 vs. A3 (C). [file FSN3-14-e72145-s001.docx]

**Table S1**  DCVCs between different planting altitudes.

| **No.** | **A4 vs. A3** | | | | |  | | **A4 vs. A2** | | | | |  | | **A4 vs. A1** | | | | |
| --- | --- | --- | --- | --- | --- | --- | --- | --- | --- | --- | --- | --- | --- | --- | --- | --- | --- | --- | --- |
|  | **Up** | | | **Down** | | **Up** | | | | | **Down** | | **Up** | | | | | **Down** | |
| 1 | Alcohol | 1,4-Butanediol | Terpenoids | | trans-Rose oxide | | Alcohol | | 1,4-Butanediol | Ketone | | 2-Heptanone | | Ketone | | Camphenone, 6- | Acid | | 2-Pentenoic acid |
| 2 | Acohol | 2-Propanol, 1,3-dichloro- | Alcohol | | 4-Hexen-1-ol, (Z)- | | Aldehyde | | Decanal | Ketone | | 3-Octanone | | Ketone | | 2-Nonen-4-one | Ketone | | 2-Heptanone |
| 3 | Aldehyde | 4-Pyridinecarboxaldehyde | Ketone | | 1-Hexen-3-one | | Aldehyde | | 4-Heptenal, (Z)- | Terpenoids | | trans-Rose oxide | | Aldehyde | | Decanal | Ketone | | 1-Hexen-3-one |
| 4 | Hydrocarbons | Cyclohexene, 4-ethenyl- | Ketone | | 2-Heptanone | | Alcohol | | 3-Octen-1-ol, (Z)- | Acid | | 5-Methylhexanoic acid | | Ester | | .delta.-Nonalactone | Ketone | | 3-Octanone |
| 5 | Ester | 2-Butanol, 3-methyl-, acetate | Ketone | | 3-Octanone | | Terpenoids | | trans-.beta.-Ocimene | Ester | | Butanoic acid, ethyl ester | | Aldehyde | | 4-Pyridinecarboxaldehyde | Alcohol | | trans-Ocimenol |
| 6 | Ester | Butanenitrile, 4-(methylthio)- | Ester | | Methyl isovalerate | | Alcohol | | 2-Propanol, 1,3-dichloro- | Acid | | 2-Furancarboxylic acid | | Hydrocarbons | | Bicyclo(3.3.1)non-2-ene | Acid | | 5-Methylhexanoic acid |
| 7 | Alcohol | Ethene, 1,1'-[oxybis(2,1-ethanediyloxy)]bis- | Ketone | | 4-Hydroxy-3-hexanone | | Ester | | Butanoic acid, 2-pentenyl ester, (Z)- | Terpenoids | | Cyclohexanol, 1-methyl-4-(1-methylethyl)-, cis- | | Heterocyclic compound | | Indene | Acid | | 2-Furancarboxylic acid |
| 8 | Ester | 3-Octenoic acid, methyl ester, (E)- | Alcohol | | Cyclopentanethiol | | Ester | | Hexanoic acid, cyclopentyl ester | Heterocyclic compound | | 1H-Imidazole, 4,5-dihydro-2-methyl- | | Alcohol | | 1,4-Butanediol | Acid | | 4-Pentenoic acid |
| 9 | Terpenoids | Naphthalene, decahydro-4a-methyl-1-methylene-7-(1-methylethylidene)-, (4aR-trans)- | Alcohol | | 4-Hexen-1-ol, (E)- | | Terpenoids | | 1,3,7-Octatriene, 3,7-dimethyl- | Terpenoids | | Cyclohexanol, 1-methyl-4-(1-methylethyl)-, trans- | | Alcohol | | 3-Octen-1-ol, (Z)- | Aromatics | | Benzene, 1,2,3,5-tetramethyl- |
| 10 | Terpenoids | 1-Isopropyl-4,7-dimethyl-1,2,3,4,5,6-hexahydronaphthalene | Ester | | Hexadecanoic acid, ethyl ester | | Ester | | 2-Nonynoic acid, methyl ester |  | |  | | Alcohol | | 2-Propanol, 1,3-dichloro- | Terpenoids | | Cyclohexanol, 1-methyl-4-(1-methylethyl)-, cis- |
| 11 | Terpenoids | 4a,8-Dimethyl-2-(prop-1-en-2-yl)-1,2,3,4,4a,5,6,7-octahydronaphthalene | Ester | | Propanoic acid, 2-methyl-, butyl ester | | Ether | | Butanenitrile, 4-(methylthio)- |  | |  | | Terpenoids | | trans-.beta.-Ocimene | Ester | | Hexadecanoic acid, ethyl ester |
| 12 | Terpenoids | Naphthalene, 1,2,4a,5,8,8a-hexahydro-4,7-dimethyl-1-(1-methylethyl)-, (1.alpha.,4a.beta.,8a.alpha.)-(.+/-.)- | Ketone | | Ethanone, 1-(4-hydroxy-3-thienyl)- | | Ester | | Pentanoic acid, 2-hydroxy-3-methyl-, methyl ester |  | |  | | Terpenoids | | 2,4,6-Octatriene, 2,6-dimethyl- | Ketone | | Ethanone, 1-(4-hydroxy-3-thienyl)- |
| 13 |  |  | Ketone | | Piperidin-3-one, 2,2,5-methyl- | | Terpenoids | | Cyclohexanol, 5-methyl-2-(1-methylethyl)- |  | |  | | Ester | | Hexanoic acid, cyclopentyl ester | Ketone | | Piperidin-3-one, 2,2,5-methyl- |
| 14 |  |  | Heterocyclic compound | | 1H-Imidazole, 4,5-dihydro-2-methyl- | | Aldehyde | | 3-Cyclohexene-1-acetaldehyde, .alpha.,4-dimethyl- |  | |  | | Terpenoids | | 1,3,7-Octatriene, 3,7-dimethyl- | Aromatics | | Benzene, 1,2,4,5-tetramethyl- |
| 15 |  |  |  | |  | | Ester | | 3-Octenoic acid, methyl ester, (E)- |  | |  | | Ether | | Benzene, (2-methoxyethyl)- | Terpenoids | | Cyclohexanol, 1-methyl-4-(1-methylethyl)-, trans- |
| 16 |  |  |  | |  | | Terpenoids | | (1Alpha,3beta,4beta)-p-menthane-3,8-diol |  | |  | | Hydrocarbons | | 1,3-Cyclohexadiene, 5-butyl- | Ester | | Propanoic acid, 2-methyl-, butyl ester |
| 17 |  |  |  | |  | | Terpenoids | | p-Menthane-3,8-diol, cis-1,3,trans-1,4- |  | |  | | Ester | | 3-(Methylthio)propanoic acid methyl ester |  | |  |
| 18 |  |  |  | |  | | Ester | | Pentanoic acid, 2-hydroxy-4-methyl-, methyl ester |  | |  | | Ether | | Butanenitrile, 4-(methylthio)- |  | |  |
| 19 |  |  |  | |  | |  | |  |  | |  | | Ester | | Pentanoic acid, 2-hydroxy-3-methyl-, methyl ester |  | |  |
| 20 |  |  |  | |  | |  | |  |  | |  | | Ester | | Butanoic acid, 2-pentenyl ester, (Z)- |  | |  |
| 21 |  |  |  | |  | |  | |  |  | |  | | Terpenoids | | p-Menthane-3,8-diol, cis-1,3,trans-1,4- |  | |  |
| 22 |  |  |  | |  | |  | |  |  | |  | | Terpenoids | | (1Alpha,3beta,4beta)-p-menthane-3,8-diol |  | |  |
| 23 |  |  |  | |  | |  | |  |  | |  | | Terpenoids | | 2,4,6-Octatriene, 2,6-dimethyl-, (E,E)- |  | |  |
| 24 |  |  |  | |  | |  | |  |  | |  | | Ester | | Pentanoic acid, 2-hydroxy-4-methyl-, methyl ester |  | |  |
| 25 |  |  |  | |  | |  | |  |  | |  | | Terpenoids | | 2,4,6-Octatriene, 2,6-dimethyl-, (E,Z)- |  | |  |
| 26 |  |  |  | |  | |  | |  |  | |  | | Aldehyde | | 3-Cyclohexene-1-acetaldehyde, .alpha.,4-dimethyl- |  | |  |

**Figure S1.** Linear discriminant analysis of bacterial and fungal communities in samples.


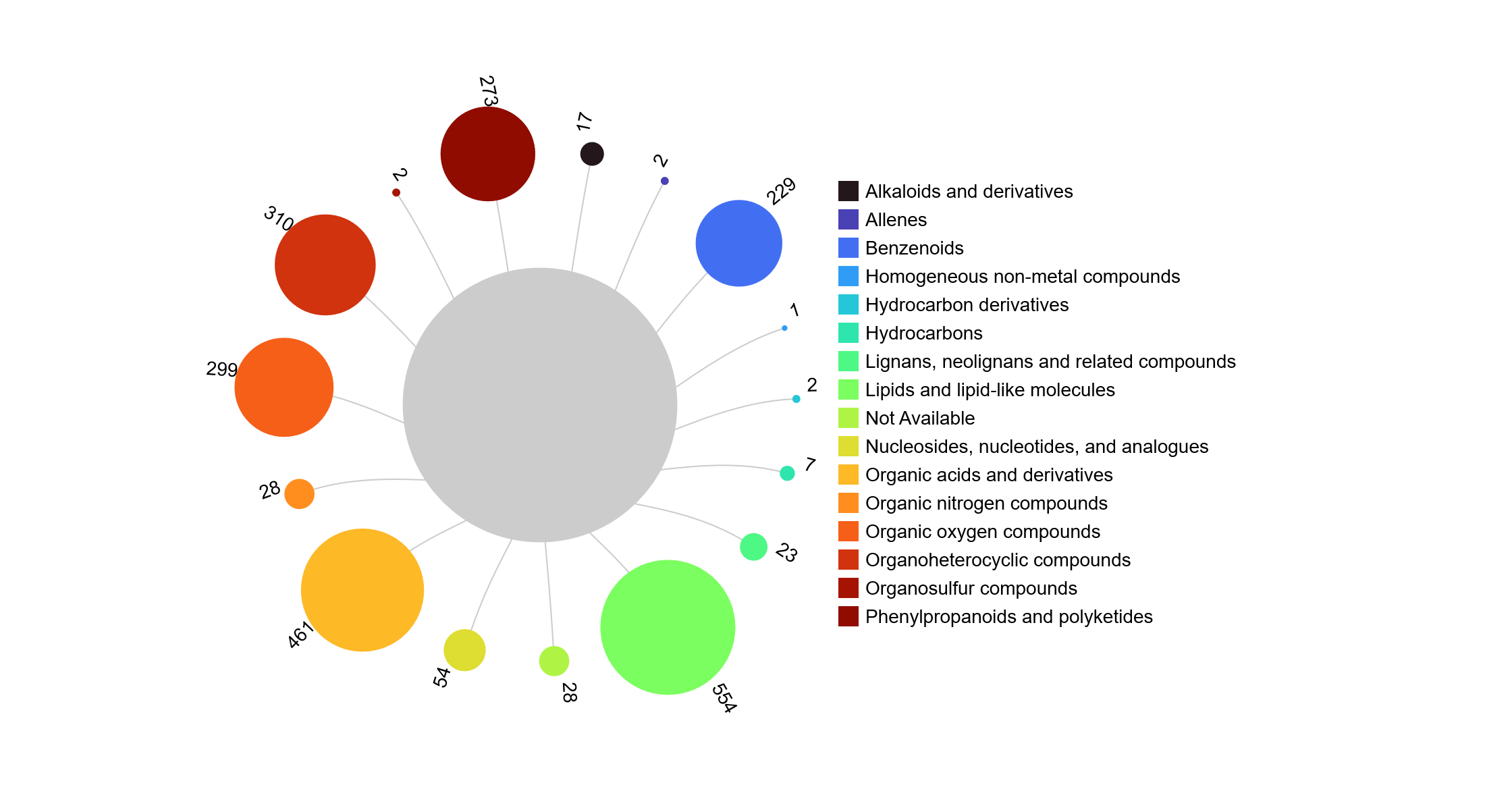


**Figure S2.** Super-classes of chemical compounds during the coffee fermentation of different planting altitudes, the different colors represented different super-classes, while the numbers indicated the number of the class within each super-class.


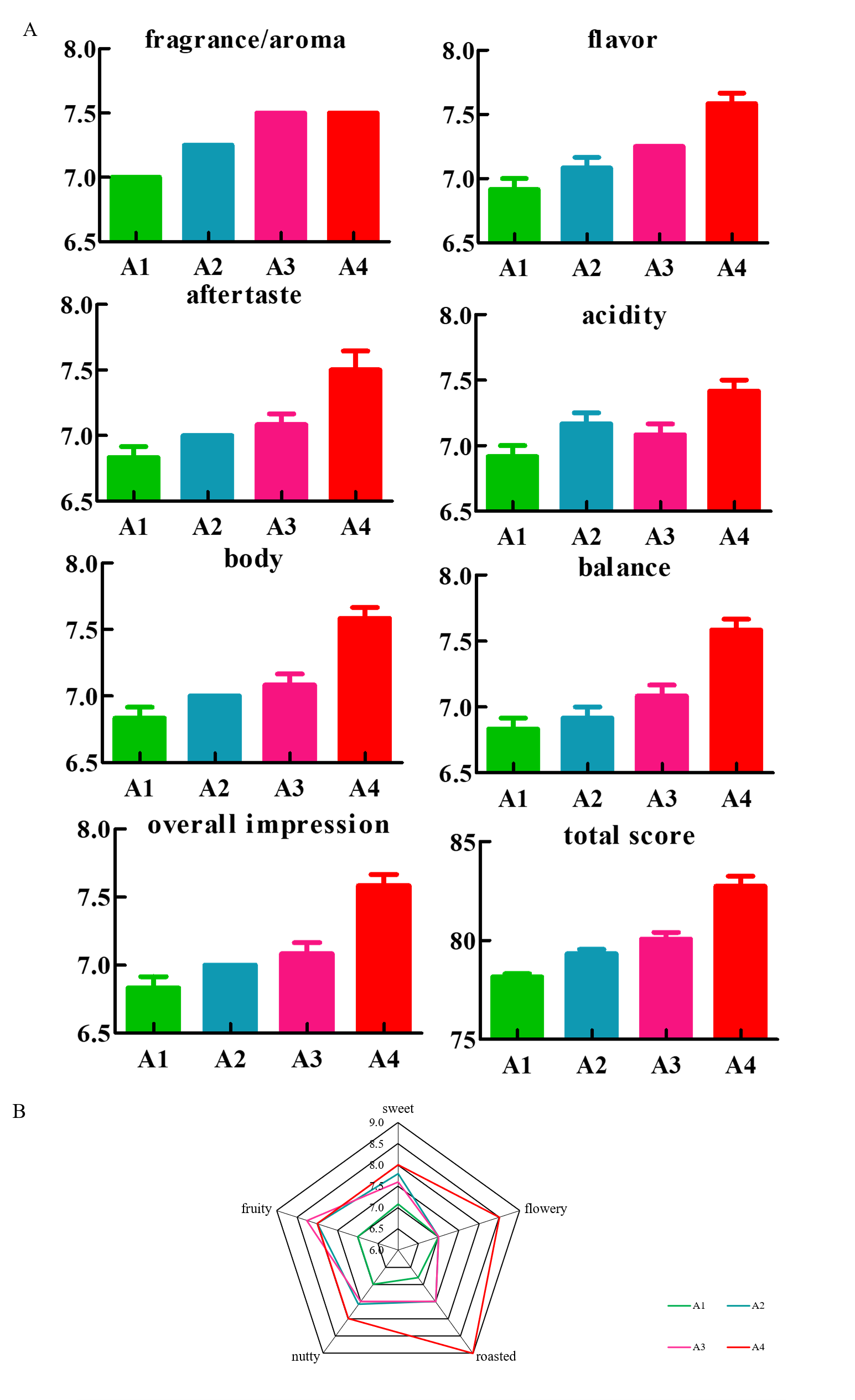


**Figure S3.** Coffee flavor characteristics of different planting altitudes, scores from the coffee cupping test (A), aroma score (B).

**Figure S4.**  The flavor characteristics of DCVCs in A4 vs. A1 (A); the flavor characteristics of DCVCs in A4 vs. A2 (B); the flavor characteristics of DCVCs in A4 vs. A3 (C).
